# Artisanal small-scale mining: Potential ecological disaster in Mzingwane District, Zimbabwe

**DOI:** 10.4102/jamba.v7i1.158

**Published:** 2015-05-28

**Authors:** Siduduziwe Ncube-Phiri, Alice Ncube, lessing Mucherera, khululi Ncube

**Affiliations:** 1 Department of Geography, Bindura University of Science Education, Zimbabwe; 2 Disaster Management Training and Education Centre for Africa, University of the Free State, South Africa; 3 Zimbabwe School of Mines, Coghlan Ave Ext, Zimbabwe

## Abstract

Artisanal small-scale mining (ASM) has devastating impacts on the environment, such as deforestation, over-stripping of overburden, burning of bushes and use of harmful chemicals like mercury. These environmental impacts are a result of destructive mining, wasteful mineral extraction and processing practices and techniques used by the artisanal small-scale miners. This paper explores the ecological problems caused by ASM in Mzingwane District, Zimbabwe. It seeks to determine the nature and extent to which the environment has been damaged by the ASM from a community perspective. Interviews, questionnaires and observations were used to collect qualitative data. Results indicated that the nature of the mining activities undertaken by unskilled and under-equipped gold panners in Mzingwane District is characterised by massive stripping of overburden and burning of bushes, leading to destruction of large tracts of land and river systems and general ecosystem disturbance. The research concluded that ASM in Mzingwane District is an ecological time bomb, stressing the need for appropriate modifications of the legal and institutional frameworks for promoting sustainable use of natural resources and mining development in Zimbabwe. Government, through the Ministry of Small Scale and Medium Enterprises, need to regularise and formalise all gold mining activities through licensing, giving permanent claims and operating permits to panners in order to recoup some of the added costs in the form of taxes. At the local level, the Mzingwane Rural District Council (MRDC) together with the Environmental Management Agency (EMA) need to design appropriate environmental education and awareness programmes targeting the local community and gold panners.

## Introduction

Mineral extraction is the most destructive industry to the environment (Chenje 2000), and artisanal small-scale mining (ASM) contributes to this destruction. The definition of ASM varies from country to country (Musingwini & Sibanda 1999), depending on variables such as investment costs, mine output, labour productivity, size of concessions, amount of resources, annual sales and levels of technology used (Meech, Veiga & Troman 1998). However, this paper defines ASM as an activity that encompasses small, medium, informal, legal and illegal miners who use rudimentary methods and processes to extract mineral resources. These miners are unskilled, lack knowledge and have little appreciation of the environment (Veiga & Hinton 2002). In this article, such mines are individual enterprises or small family-owned companies not affiliated to multinational companies.

Artisanal mining sustains the livelihood of at least two million people in Zimbabwe directly and indirectly through ancillary services and secondary economic activities (Maponga & Ngorima 2003). However, its overbearingly negative environmental impacts outweigh the socioeconomic benefits. Air pollution, water pollution and land degradation are common in areas where such mining is carried out. ASM operations feature a number of rudimentary practices that pollute the air and contaminate resident water bodies and soils, for example through the use of mercury (Hilson & Van der Vorst 2002). Mercury is used for gold amalgamation in artisanal mining. The amalgamation process transforms elemental mercury into methyl mercury (United Nations Environment Programme [UNEP] 2002), a toxic organic compound which poses a threat to the health of animals, humans (International Council for Science 2007) and aquatic life (Tunhuma 2007).

Van Straaten (2000) notes that mining activities involve digging in river banks and river beds, causing erosion as well as siltation as large amounts of sediment feed into the river system (Pereira 2009). Thus, the activities involved in ASM, if uncontrolled, have the potential to turn the hazard into an ecological disaster.

## Background

Mzingwane District in Matabeleland South Province of Zimbabwe has for the past two decades experienced a sharp decline in subsistence agriculture as a result of unemployment and recurring droughts (Taylor 1998). This phenomenon has forced many households to diversify into gold mining along the Insiza and Umzingwane rivers and in disused mines (Kamete 2007). In the process of diversification, Cunningham *et al*. (2005) cited by Ncube et al. (2010:80) view ‘the poor as both the victims and agents of environmental degradation forced to engage in unsustainable activities to meet short-term survival needs’. Dreschler (2001) estimated that the number of people deriving their livelihood from artisanal mining in Zimbabwe could be well over 2 million, from service providers to panners. Artisanal mining has received a boost in Zimbabwe in the last decade because of new government policies, through the Ministry of Small Scale and Medium Enterprises, that promote small-scale mining as miners are encouraged to peg claims and operate legally. However, such policies have overlooked key issues, such as equipping and training small-scale miners in a bid to help minimise the adverse impacts on the environment.

Diversification into artisanal small-scale gold mining in this district, whilst providing employment and livelihoods to many, pose ecological challenges. Artisanal gold mining in Mzingwane District depends heavily on water for panning processes. The water challenges are made worse by the fact that the district is a water catchment area for Bulawayo Metropolitan Province, Esigodini Rural District, Mawabeni District centre of development and surrounding areas (Ncube et al. 2010). According to Chiwawa (1998), the long-term sustainability of a catchment area and its rivers highly depends on the type and volume of economic activity taking place within the catchment area. Five dams supply water to these areas, namely Umzingwane, Upper Ncema, Lower Ncema, Nyankuni and Mtshabezi. According to the Food and Agriculture Organisation of the United Nations (FAO) (2004), the dams are along three major rivers – Umzingwane, Insiza and Mtshabezi – where alluvial gold panning activities are concentrated. The river system has been identified as a key variable negatively affected by gold panning. Drying up of dams and rivers in Mzingwane District has been blamed largely on siltation (Zimbabwe National Water Authority [ZINWA] 2009). Siltation of rivers ‘reduces river conveyance and the storage capacity of reservoirs, which in turn will make several areas prone to flooding’ (Shoko 2002:1).

Gold panning processes on the river banks, beds and the surrounding areas discharge huge amounts of loose silt and heavy metals into the river system. Eventually these are washed into the dams, increasing the risk of siltation, flooding and drying up of water reservoirs (Shoko 2002). The total capacity of the 2168 dams in the Zimbabwean part of the Limpopo basin, of which Umzingwane, Upper Ncema, Lower Ncema, Inyankuni and Mtshabezi dams are a part, has fallen by about 29 million m^3^ as a result of siltation (FAO 2004). The effect on the storage capacity has already been felt in and around the district. Bulawayo is the worst hit by water shortages as a result of reduced storage capacity of the dams supplying water to the city. Bulawayo's water consumption stands between 134 000 m^3^ and 140 000 m^3^, of which 58% come from the Umzingwane catchment area (Bulawayo City Council 2011).

## Study setting

Mzingwane District is one of six districts in the drought-stricken Matabeleland South Province of Zimbabwe. The area receives erratic rainfall averaging 760 mm per annum. The district covers a surface area of 2820 km^2^ and constitutes 20 wards with a total population estimated at 58 569. Artisanal small-scale gold mining occupies approximately 20% of the total surface area, which is 564 km^2^ (Dreschler 2001). Most people in this area have diversified to gold panning to sustain their livelihoods, making the area more vulnerable to associated risks. Land degradation experienced in the Umzingwane and Insiza rivers caused siltation of the associated dams, thereby affecting storage capacity, water quality of these dams and supply of water in Bulawayo Metropolitan.

**FIGURE 1:**
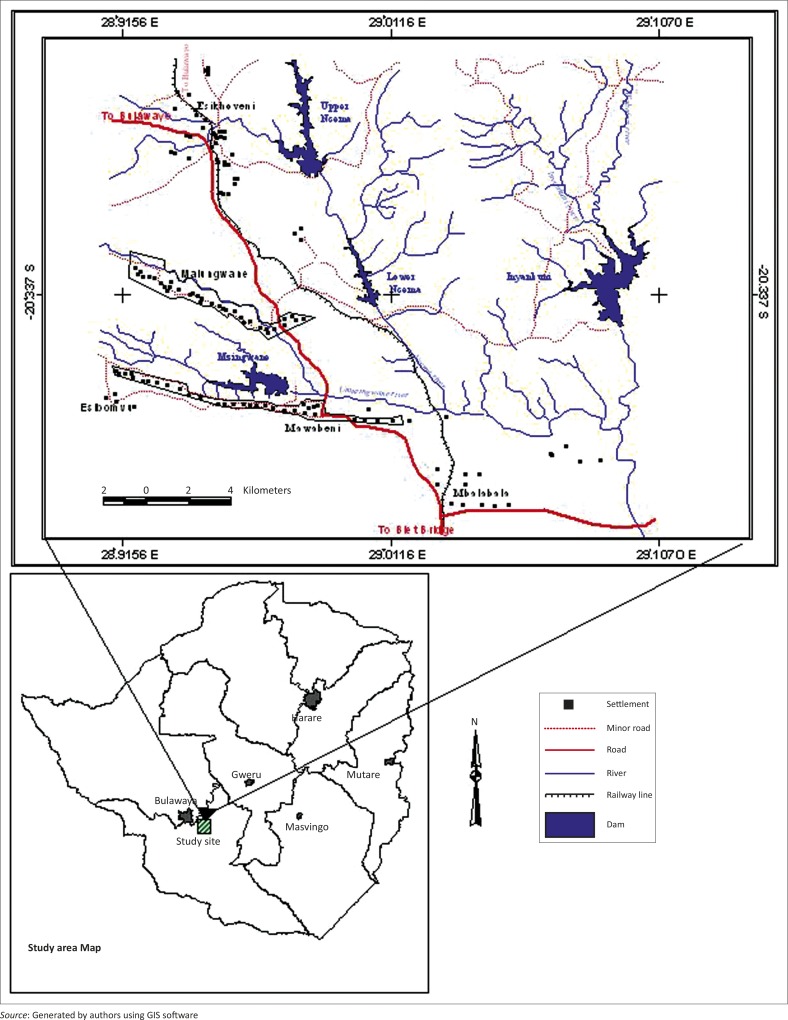
Mzingwane District study area.

*Source:* Generated by authors using GIS software

A large proportion of the catchment area for the dams falls within this area (Figure 1) with major dams such as Umzingwane, Upper Ncema, Lower Ncema, Inyankuni and Mtshabezi under threat. A lot of alluvial gold panning activities take place on the river beds and banks of the Umzingwane and Mtshabezi.

## Research objectives

The objectives of this paper are to:

- provide a framework for the understanding of environmental problems associated with ASM in Mzingwane District
- determine the impact of artisanal small-scale gold mining on the Mzingwane district ecosystem
- quantify the surface area affected by panning activities
- raise community awareness of the possible hazards associated with ASM.

## Justification

Zimbabwe has not paid enough attention to the impact of mercury contamination and other environmental challenges as a result of artisanal gold mining activities. There are various possible reasons. Firstly, there is no state-of-the-art equipment such as the ‘ultra-clean free-metal sampling protocol’ (Gill & Fitzgerald 1985) to be used in carrying out studies to predict the potential impacts of mercury poisoning on humans and aquatic life. Secondly, Zimbabwe is trying to recover from its inflationary period and mining seems to be taking the centre stage in development processes (Taylor 1998). This paper therefore seeks to provide a foundation for a better understanding of the nature and degree of environmental problems in Mzingwane District as a result of ASM so that environmentally friendly gold mining practices can be promoted in the Mzingwane study area. Over time, the research will equip the communities involved and make them more resilient to hazards.

### Literature review

#### Impact of artisanal small-scale mining

Artisanal small-scale mining (ASM) operations feature a number of rudimentary practices that pollute air and contaminate resident water bodies and soils, for example through the use of mercury (Hilson & Van der Vorst 2002). Mercury is used for gold amalgamation in artisanal mining. The amalgamation process transforms elemental mercury into methyl mercury (United Nations Environment Programme [UNEP] 2002). Methyl mercury, one of the most toxic organic compounds, is a powerful neurotoxin that works its way up the food chain through bioaccumulation. This poses a threat to the health of animals, humans (International Council for Science 2007) and aquatic life (Tunhuma 2007). Mercury is also poisonous when inhaled (Larceda 1997), as it causes lung cancer and skin disease. The mercury used by panners is also discharged into ecosystems in an abusive manner (Pfeiffer & Larceda 1988; Meech et al. 1998).

According to Shoko (2002), problems caused by the clearing of land include soil erosion, siltation, soil compaction, destruction of ecosystems and loss of biodiversity. Water pollution causes the destruction of aquatic ecosystems, plant life and depletion of fresh water resources. For Babut et al. (2005) and Hinton, Veiga and Veiga (2003), the effect of artisanal small-scale gold mining on the ecology is the fragmentation of ecosystems and habitats, obstructing migratory routes to breeding and feeding grounds used by wildlife, and depletion of fisheries. Miththapala (2008) argues that land degradation leads to loss of livelihoods and reduced food security. Shoko (2002) also lists problems caused by air pollution, such as ozone depletion and global warming, in which greenhouse gases trap long-wave radiation, thereby increasing the temperature on the earth's surface. Noise pollution from stamp mills, pan dishes and blasting also causes ill health, loss of hearing and migration of wildlife and birds. Land degradation also results in the loss of aesthetic value of the landscape as mining activities leaves open pits and mounds of sand.

#### Hazard and risk mitigation

Establishing sound knowledge systems of hazards and risks associated with small-scale mining helps communities develop relevant coping strategies (Wisner et al. 2004). An understanding of the ecological disasters associated with gold panning is critical to decision-making, planning and implementation of development projects that are competing for the same resources in the district, for example rural, urban, legal mining, illegal mining, and irrigated commercial and subsistence agriculture. This research therefore provides a step towards good land management practices, crucial in sustainable utilisation of natural resources. The paper also contributes to the fundamentals of formalising illegal gold mining and promoting community participation in policy making and environmental protection, as the same community is involved in these illicit activities. Community participation helps promote efforts that ‘advocate for cleaner production techniques to be used in the purification of gold to reduce impacts on gold panners and environment’ (Ghose 2003:169).

## Methodology

### Data collection

A qualitative research methodology was used to collect data on the perceptions of the ecological impact of gold panning in Mzingwane District. The research tools used included interviews, questionnaires, field observations, photo visioning and a review of related literature. Data were collected using a pre-tested questionnaire distributed to 206 randomly selected households in three villages from a population of 1370 households (15% of the population). It was not possible to involve everyone because of time and financial constraints. The questionnaire standardised and ensured uniformity in the nature of the data and perceptions of individuals on the ecological impacts. Key informant interviews were held with nine heads of departments. These included the heads of departments in local government, the Ministry of Mines and Mining Development (MMMD), the Mzingwane Rural District Council (MRDC), Bulawayo City Council (BCC), ZINWA, National Parks and Wildlife (NPW) and the Environmental Management Agency (EMA). Seven traditional leaders and three local councillors were strategically targeted. The respondents were coded using a numbering system to protect their identity. Focus group discussions using semi-structured interviews were held with groups found at panning sites. Geographical information system (GIS) was used to represent the spatial relationship of phenomena. Field visits were also carried out for ground truth verification for data validation. Pictures of burnt areas and degraded land were taken for analysis.

### Sampling

Purposive sampling was used to select three villages that are at the hub of panning activities, namely Mzinyathini, Malungwane and Mawabeni. Purposive sampling is selecting a sample based upon the researcher's judgement and specific purpose rather than randomly (Teddlie & Yu 2007). As suggested by Teddlie and Yu (2007), purposive sampling allowed for ease of access to artisanal small-scale miners and household heads. The study also had to be specific in selecting the heads of departments as well as traditional leadership structures earmarked for this research. These departments are in charge of environmental issues and are the custodians of the information that the research focused on. The research assumed that artisanal small-scale miners had similar traits and circumstances and each one of them was believed to represent those involved in artisanal gold mining in the district.

## Findings and discussion

This section presents the main findings of the research as well as some of the themes that emerged. One hundred and twenty one households (58.7%) of the sampled population were engaged in ASM. The core themes addressed include demographic information of respondents, drivers of gold panning and mining practices, negative impacts of gold mining on the environment, elements at risk, control measures put in place as well as potential ecological disasters.

### Demographics and nature of panning

**FIGURE 2:**
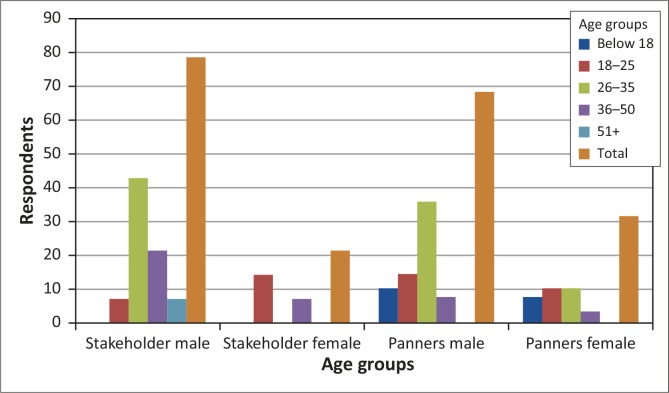
Respondents’ age profile.

The age group 26–35 years constituted the highest number of panners (45%), followed by the age group 18–25 years (25%) and lastly the age groups below 18 and 51 + (Figure 2). There were more male (68%) than female (32%) panners. Women's roles were sieving and preparing food. In total, 79% of the respondents were men whilst 21% were women. Panners represented the economically active age group (18–50 years) who are out of formal employment.

The majority of panners have a primary (64%) and secondary (32%) level of education (Table 1). Bhebhe (2009) argues that most children, especially boys, drop out of school to venture into gold panning and therefore have limited livelihood options. Formal employment demands higher qualification than primary and secondary education. These panners would have failed to widen their employment opportunity base.

**TABLE 1:** Level of education.

Schooling	Panners	Stakeholders
No schooling	0	0
Primary level	64	30
Secondary level	32	60
Tertiary level	4	10

High percentages of panners were engaged in full-time (26%) and part-time gold panning (51%) (Table 2). A large number of panners were involved in panning during the period 2000–2011 (Table 3), which coincides with Zimbabwe's massive economic meltdown. The numbers rose in the years 2005–2008, when the economic situation in Zimbabwe had deteriorated to unacceptable levels.

**TABLE 2:** Panners’ nature of engagement.

Nature of engagement	Panners (%)
Full time	26
Part time	51
Seasonally	23
Occasionally	0

**TABLE 3:** Panners’ experience.

Panning experience (years)	Panners (%)
0–5	33
6–10	41
11–15	17
16–20	9
20+	0

The main reasons driving respondents to panning are lack of employment (90% of panners), inadequate income (60%) and drought (40%) (Table 4). These findings are confirmed by Lungu and Shikwe (2007), who found that the need for income force most people into this dangerous venture.

**TABLE 4:** Panning drivers.

Panning drivers	Panners (%)
Lack of employment	90
Inadequate income	60
Drought	40

### Environmental problems of artisanal small-scale mining

**FIGURE 3:**
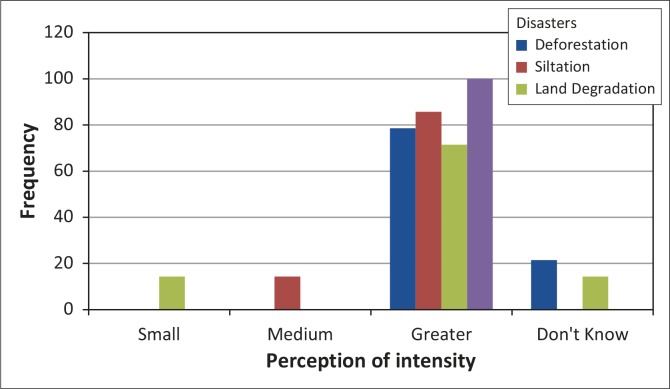
Respondents’ perception of intensity of environmental problems.

In all three study areas respondents were aware of the negative impact of gold panning. Whilst it is a lucrative venture, respondents identified specific challenges associated with ASM, including epidemics, gullies, unsafe pits, siltation, land degradation, deforestation and veld fires. Triangulated data on the extent of ecological problems from both panners’ and stakeholders’ perspectives indicate a high level of awareness of negative environmental impacts of gold panning (Figure 3).

**FIGURE 4:**
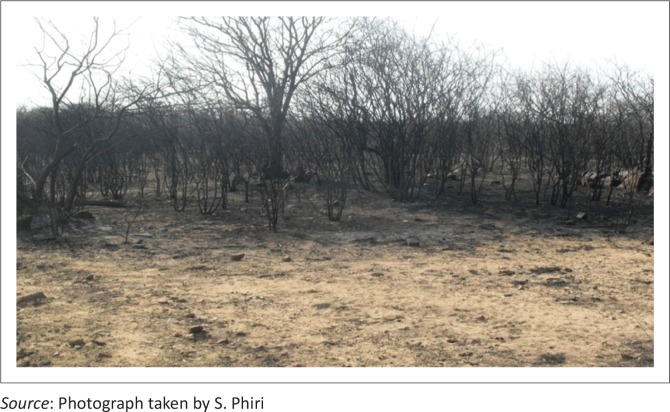
Ward 20 severely burnt by high-profile, mechanised panners.

*Source*: Photograph taken by S. Phiri

Veld fires were ranked as having the greatest extent (100%), followed by siltation (84%), deforestation (78%) and land degradation (65%). Community members were concerned that fire hazards resulting from gold panning activities had become a serious threat, causing widespread damage to the local environment. It emerged that almost three-quarters of the district had been burnt by artisanal small-scale miners exploring for gold using metal detectors. Communities expressed displeasure at the environmentally unfriendly activities and pointed out that grazing areas were burnt and cattle had nothing to feed on, thus local livelihoods have been compromised in the process. Ward 20, for example, was severely damaged in a veld fire, leaving livestock without grazing land (Figure 4). Livelihoods were at stake because of the wish to make a ‘quick buck’ from gold panning, a non-renewable resource.

**FIGURE 5:**
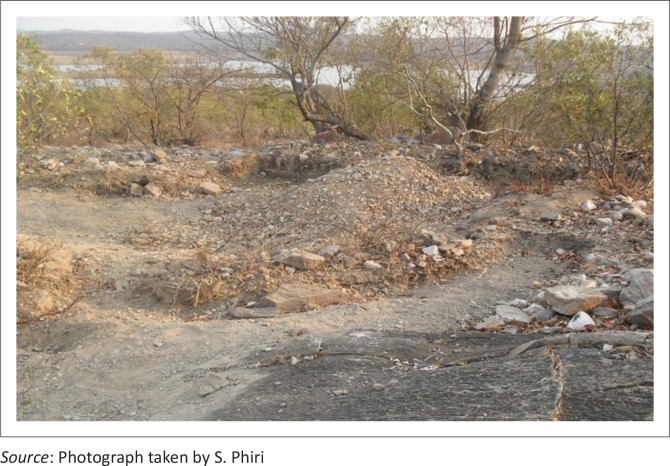
Panning activities near Umzingwane Dam.

*Source*: Photograph taken by S. Phiri

**FIGURE 6:**
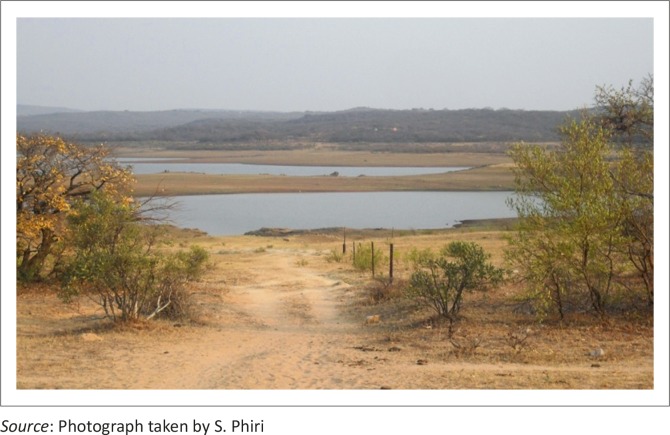
Umzingwane Dam, central section.

*Source*: Photograph taken by S. Phiri

The levels of dams (Table 5) are affected by panning activities which are carried out near water sources (Figures 5 and 6). There was great concern amongst respondents about the water situation because gold panning practices were deemed to affect water quality and quantity in local dams. The central part of Umzingwane Dam is partly silted, with an island of silt at the centre (Figure 6). Observations indicated that the dam cannot survive siltation as artisanal small-scale miners are mining too close to the dam (Figure 5).

**TABLE 5:** Catchment dam levels.

Dams	Years			
	2008 (%)	2009 (%)	2010 (%)	2011 (%)
Umzingwane	19	24	7	15
Inyankuni	14	8	0	20
Upper Ncema	45	65	0	30
Lower Ncema	63	73	51	76

*Source:* ZINWA (2008, 2009, 2010, 2011)

Note: Please see the full reference list of the article, Ncube-Phiri, S., Ncube, A., Mucherera, B. & Ncube, M., 2015, ‘Artisanal small-scale mining: Potential ecological disaster in Mzingwane District, Zimbabwe’, *Jàmbá: Journal of Disaster Risk Studies* 7(1), Art. #158, 11 pages. http://dx.doi.org/10.4102/jamba.v7i1.158, for more information.

One key informant expressed the need to scoop dams because some had never been scooped since their construction in 1942 (Upper Ncema and Lower Ncema) and the latest in 1973. Turbidity was another issue raised by a key informant from the BCC, who lamented the negative impact of artisanal gold mining on water quality. He noted that the impact manifested as costs incurred by dam maintenance and water purification. Chiwawa (1998) argues that degradation caused by small-scale miners contributes largely to water pollution and depletion yet is rarely talked about. Water from Inyakuni, Upper Ncema and Lower Ncema was said to be muddy, consuming huge quantities of purification chemicals, and as a result the BCC drew most of its water from Umzingwane Dam. Although the respondents could not share figures, they reiterated the high cost of water purification. That information was said to be highly classified. The costs, however, led the BCC to concentrate mostly on Umzingwane Dam because it was cheaper to purify its water.

Large water quantities at the end of the season at Upper Ncema and Lower Ncema (Table 5) illustrate the BCC's overdependence on Umzingwane Dam. The discussion indicated that 80% of water from Umzingwane Dam was drawn by the BCC yet the dam was not spared from siltation. Mining activities upstream interfered with the river system, thereby reducing the water flow rate, thus denying communities downstream adequate water for their livelihoods. Mpendazoe (1996) notes that large earth heaps on the river bed disturbs the natural water flow. Respondents reiterated that Sheet Dam in the same district was fully silted and washed out. The ZINWA reports for the period 2008–2011 (Table 5) on dam levels had even left it out of consideration. It used to have a full supply capacity of 1 169 000 m^3^ of water before siltation. However, the cost of its maintenance was reported to have escalated greatly.

Observations made at the panning sites established land degradation as another environmental challenge. Earth heaps of overburden from massive excavations were found in all three study areas.

**FIGURE 7:**
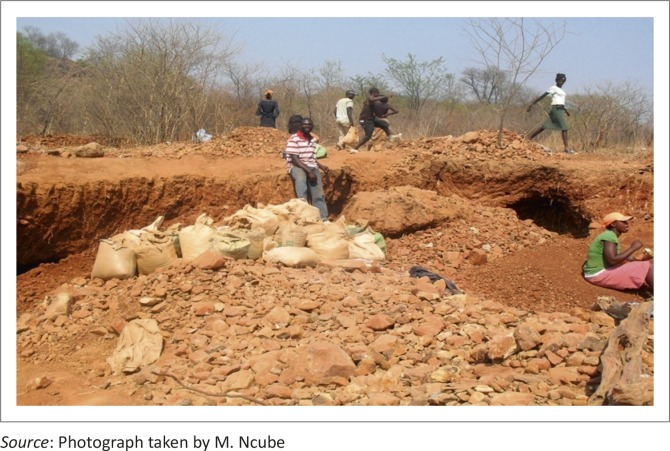
Excavations and land degradation in Mawabeni.

*Source*: Photograph taken by M. Ncube

The ASM area Mawabeni 1 covers about 1000 m x 200 m or 0.2 km^2^ (Table 6). It emerged from focus group discussions held with groups found on panning sites that, on average, the panners moved 4000 kg of soil per person daily. Figure 7 shows bags full of soil, confirming the information obtained from focus group discussions that panners move 44 wheelbarrows or 80 x 50 kg bags, which are equivalent to four tonnes of soil every day. As their working day is approximately 14 hours according to focus group discussions and there were 43 gold panners found at the site, it could be calculated that 172 000 kg of soil is moved by panners every day and processed in the water along Umzingwane River, which then carries the residue downstream. The focus group discussion further disclosed that panners obtained 0.2 g of gold per tonne on average. It emerged that they sell their gold for $30/g to illegal gold buyers, against Reserve Bank of Zimbabwe regulations. The cost of mining impacts does not match the income received. According to the Association of African Universities (2008), a woman in Ward 1 of Mzingwane District reported to be destitute after allowing gold panners on her plot. The plot was completely destroyed, depriving her of the best land for cultivating crops that she had ever owned.

**TABLE 6:** Artisanal small-scale mining areas.

Village	m^2^	Total m^2^	Total km^2^
Malungwane	3000 m x 200 m	600 000 m^2^	0.6 km^2^
Mawabeni 1	1000 m x 200 m	200 000 m^2^	0.2 km^2^
Mawabeni 2	5000 m x 3000 m	15 000 000 m^2^	15 km^2^
**Total surface area excavated**	**-**	**15 800 000 m^2^**	**15.8 km^2^**

Mzingwane District covers 2820 km^2^, of which 20% is occupied by gold panning (Dreschler 2001), which means that gold panning occupies only 564 km^2^. The observed surface area used for ASM, according to observations carried out for this research, is 15.8 km^2^ (Table 6), covering 2.8% of the total panning area of 20% put forward by Dreschler (2001). The observed panning area is 0.56% of Mzingwane district's total area.

**FIGURE 8:**
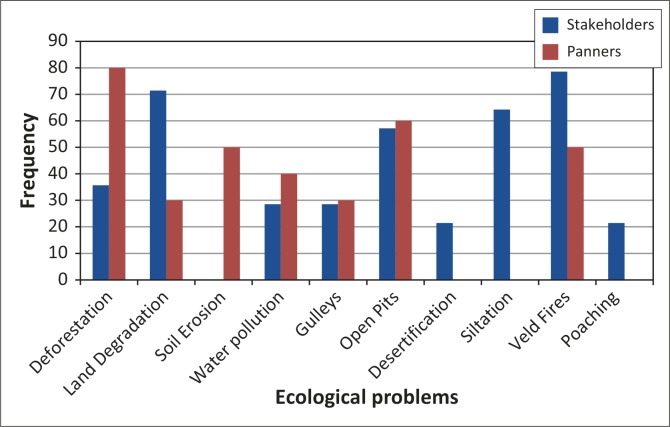
Ecological problems in Mzingwane District identified by respondents.

Panning activities remain a major threat to the environment in Mzingwane District and continues to increase the threat to biodiversity. For stakeholders, siltation, veld fires, land degradation, unsafe pits and deforestation were the most prevalent ecological problems, whilst epidemics, gullies, pollution desertification and chemical contamination were ranked low (Figure 8). These hazards are of concern because they are a threat to the environment.

Stakeholders and artisanal small-scale miners ranked ecological problems differently (Table 7). The miners are concerned with immediate problems such as deforestation, excavations and veld fires because those are the very first activities they engage in when panning. The stakeholders, on the other hand, are concerned with long-term and secondary problems that are a result of the risk accumulation process, such as siltation and land degradation. The responses indicate that both parties are aware of the ecological impact of gold panning in the district.

**TABLE 7:** Ranking of ecological problems.

Respondents	Rank	Ecological problems	%
Stakeholders	1	Veld fires	78
	2	Land degradation	71
	3	Siltation	64
	4	Open pits	57
	5	Deforestation	35
	6	Water pollution and gully formation	28
	7	Desertification and poaching	21
Panners	1	Deforestation	80
	2	Open pits	60
	3	Veld fires and soil erosion	50
	4	Water pollution	40
	5	Land degradation and gully formation	30
	6	Desertification	0
	7	Siltation	0

As for the disposal of chemicals, miners vowed that mercury was like gold to them because it was expensive to acquire and thus they recovered all of it for reuse. The only danger could be that of accidental spilling into the water system. They assured the researchers that the hazard of mercury was contained. This is supported by an informant from the BCC, who noted that mercury levels were still within acceptable levels. However, exact figures were not available as those were highly classified.

### Elements at risk

**FIGURE 9:**
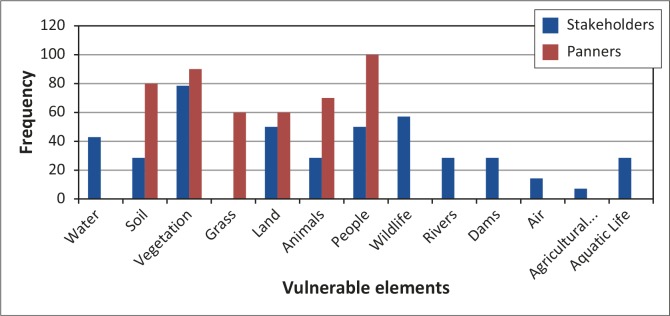
Vulnerable elements in Mzingwane District identified by respondents.

Artisanal small-scale miners and stakeholders were not only aware of the problems brought about by ASM, but were able to identify vulnerable elements at risk from panning activities. The elements identified included water, soil, vegetation, grass, land, animals, people, wildlife, rivers, dams, air, agricultural activities and aquatic life. Most panners identified people as elements at high risk, whilst stakeholders identified vegetation as highly vulnerable to panning activities (Figure 9).

The overall distribution of elements considered as the most vulnerable is indicated by the percentage frequency (Figure 9) as provided in questionnaires by both stakeholders and artisanal small-scale miners. Elements like vegetation, land, soil, animals and people were mentioned by both parties because the activities result in the fragmentation of ecosystems. It emerged that wildlife was greatly affected as animals fell into open pits that were left unattended by gold panners. The breeding places of wildlife species had also been destroyed by veld fires caused by ASM. Stakeholders listed water (40%), wildlife (58%), rivers (30%), dams (30%), air (14%), agricultural activities (8%) and aquatic life (30%) as high-risk elements whilst miners listed grass (60%), people (100%), vegetation (90%), soil (80%), land (60%) and animals (70%) as high-risk elements. Musingwini and Sibanda (1999) argue that because of their close contact to the environment panners are aware of the immediate impacts of their activities, such as death, injuries and land degradation. It emerged from stakeholders that one could not talk about wildlife outside its habitat, and thus trees, grass and soil were the major elements that needed protection from gold mining activities. Deforestation, veld fires and excavations, which affect trees, grass and soil as well as people, had long-term effects. Stakeholders’ main concern was the indiscriminate cutting down of trees, veld fires and land degradation as it negatively impacts the sustainable use of resources.

### Protection of vulnerable elements

Research indicated that some effort was made in Mzingwane to mitigate the destructive activities of gold panners (Table 8). These included awareness campaigns, policing and fines. However, respondents lamented that structures put in place to protect the environment were ineffective and sometimes even irrelevant. Corruption was cited as one of the major challenges weakening some of the efforts meant to curb pollution, environmental degradation and related challenges. It also emerged that although campaigns and awareness raising initiatives could be helpful if intensified, responsible authorities and departments were under-resourced to efficiently carry out their duties. Various stakeholders, such as the Forestry Commission, the NPW Department and ZINWA, noted that the fragmentation of policies meant to protect the environment was a challenge to environmental management. This point is supported by Spong, Booth and Walmsley (2003), who note that the fact that some policies are housed in different ministries could cause a conflict of interests.

Table 8 reveals interesting facts as far as land management in Zimbabwe is concerned. For instance, the Forestry Commission, the NPW Department, Ministry of Mines and ZINWA are supposed to carry out awareness campaigns. However, these organs have conflicting interests and as a result the campaigns are futile. The Ministry of Mines, through the Ministry of Small Scale and Medium Enterprises, is regulating and formalising all gold mining activities in Zimbabwe, promoting land degradation. On the other hand, the EMA and the Forestry Commission are against land degradation and deforestation. The law enforcers, the Zimbabwe Republic Police (ZRP), and the EMA have a challenge in discharging their mandate as they seem to be disregarding a government directive. Taking into account that the Zimbabwean economy is driven by mining, lack of coordination amongst these organs present a challenge in the implementation of awareness campaigns. Similarly there is no clearly defined boundary between ZINWA and BCC regarding water management and supply. It emerged from the study that they duplicate roles and even are in conflict in certain instances.

**TABLE 8:** Mitigation strategies in Mzingwane.

Mitigation strategies	Regulations	Regulatory authorities
Awareness campaigns	Rural District Council conservation by laws	EMA, Forestry Commission, National Parks and Wildlife Department, Ministry of Mines
Council rangers	Government and council policies	Bulawayo City Council Water Supplies, ZINWA
Policing	Statutory Instruments	ZRP
Fines	Ministry of Mines and Minerals Act	EMA

EMA, Environmental Management Agency; ZINWA, Zimbabwe National Water Authority; ZRP, Zimbabwe Republic Police.

### Potential disasters in Mzingwane District

Disasters are a function of the risk accumulation process arising from a combination of hazards, conditions of vulnerability and inability to cope with the negative consequences of risk. According to the United Nations Office for Disaster Risk Reduction (UNISDR) (2009) a disaster is:

a serious disruption of the functioning of a community or a society causing widespread human, material, economic or environmental losses which exceed the ability of the affected community or society to cope using its own resources. (p. 19)

**FIGURE 10:**
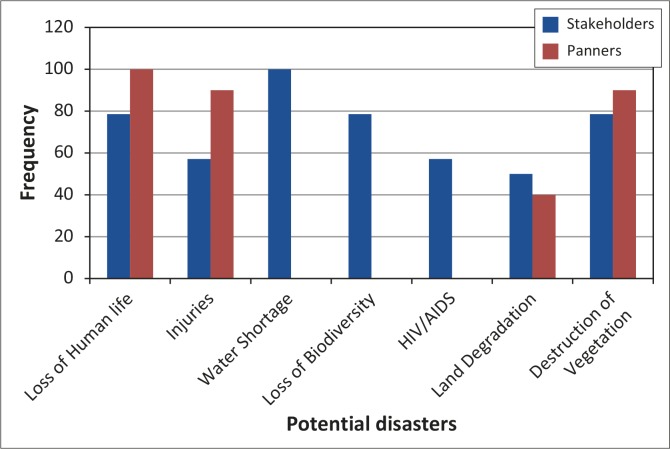
Potential disasters in Mzingwane District as perceived by respondents.

This means that a hazard impact, if met with the necessary conditions, can result in huge disturbances of the normal operations of a community, causing economic, material and environmental losses of great magnitude, making it difficult for such a community to recover without external assistance. Figure 10 summarises potential disasters in Mzingwane District, as perceived by respondents.

Although beyond the scope of this article, social disaster – loss of human life (78%), injuries (57%) and HIV and AIDS (57%) – was ranked by stakeholders as having high potential. However, they ranked water shortage (100%) highest. Panners ranked loss of human life (100%), destruction of vegetation (90%) and injuries (90%) highest. This is because panners are directly affected by deaths and injuries. In November 1992, more than 20 gold panners along Umzingwane River drowned when it rained upstream. It also emerged during discussions that 20 panners were buried along the Umzingwane River in 2010 when it rained upstream. Panners indicated that they had recently buried a fellow panner who died as a result of shaft collapse. Similarly, it emerged that in July 2011 a family of four perished as a result of shaft collapse. The data from these interviews explain the magnitude of loss of human lives and injuries. The MRDC officials expressed concern about the costs incurred by the council when burying people without relatives who died as a result of shaft collapse.

It emerged from the study that environmental health problems, especially as a result of fire and unsafe mining operations, are on the increase in Mzingwane. Respondents noted that animals have suffered burns and deaths as a result of veld fires instituted by panners to clear land for easy mineral detection by metal detectors. Although not yet established, respondents feared that ASM activities could result in long-term health problems in both animals and humans. Donkor et al. (2006) argue that gold panning could result in increased mortality as a result of respiratory and cardiovascular disease. Suspected poisoning of both wildlife and domestic animals as well as humans as a result of toxic concentrations of elements such as mercury has been reported in Mtshabezi Dam, which lies south of Esigodini in Mzingwane District. Love (1997) contends that careless handling of mercury and cyanide may lead to poisoning of water bodies. Land degradation, loss of biodiversity and water shortage emerged as major concerns in Mzingwane amongst stakeholders (Figure 10). They lamented that the area faces serious threats to the environment if the environmentally unfriendly activities continue unabated.

## Conclusion

In light of the foregoing discussion, it is evident that ASM in Mzingwane District poses a serious threat to the ecology, which in turn jeopardises human lives and livelihoods. The proliferation of illegal gold panning is likely to result in serious damage to aquatic life, biodiversity and riverine ecosystems. The loss of habitat (drying up of rivers and surface waters, degraded land) has negatively affected aquatic life, terrestrial biodiversity and productivity of both livestock and crops. If the environmental situation in the district remains unchecked, the area is heading for untold ecological disasters involving ecosystem destruction and loss of biodiversity as a result of the numerous hazards caused by ASM in search of income and livelihood. For example, according to Zimbabwe's Fourth National Report to the Convention on Biological Diversity (Ministry of Environment and Natural Resources Management 2010), fish species and populations of aquatic life in dams in the Umzingwane catchment area are reduced in numbers.

ASM activities could lead to the extinction of plant and animal species, resulting in the disruption of the ecosystem and causing an imbalance in beneficial macro- and microorganisms. The cascading effects of ecological disasters such as land degradation, loss of biodiversity, water pollution, epidemics, veld fires and desertification may appear insignificant to some populations, but their cumulative effect needs to be mitigated in order to reduce their impact on Mzingwane District and the Zimbabwean community as a whole.

Lack of employment, drought and limited livelihood options in the district and the country as a whole are the drivers of illicit panning activities in the district. The local community, ZINWA and the BCC are the worst affected by ASM activities, which cause land degradation and siltation of dams. This has an effect on ZINWA and BCC budgets because of the increased cost of water purification caused by turbidity. The article concludes that lack of effective mitigation measures exposes the district to high risks to future ecological disasters.

### Recommendations

Banning artisanal gold mining is not a viable solution in Mzingwane District because it currently is a significant livelihood option available to the local people. The article therefore recommends measures that can be adopted to reduce disaster risk.

Firstly, there is need for government to formulate a clear a policy aimed at mainstreaming disaster risk reduction in all ASM activities. This requires a collaborative effort amongst key ministries and stakeholders, including the local community. As Blackman (2002:21) stresses, for any project to be sustainable, all stakeholders need to be involved. Such a policy needs to recognise that panners are victims too and not only unruly elements, so that they receive due assistance, as suggested by Kambani (2003).

Secondly, it is necessary for the MRDC together with the EMA to design environmental education and awareness programmes targeting the local community and gold panners. Panners need to be made aware of the effects of their activities and the need for healthy ecosystems. Mpendazoe (1996) notes that mining requires a skill for sustainable operations; therefore, local leadership needs to take it upon itself to organise training workshops for artisanal small-scale miners in order to reduce associated disaster risks.

Thirdly, land rehabilitation is crucial to reduce land degradation and ecosystem disruptions; therefore, miners have to backfill their excavations. This will help to prevent wildlife and livestock from falling into pits. Taxes and fines paid by offenders should be channelled into projects that seek to mitigate water pollution, deforestation and land degradation.

Finally, it is imperative to regulate and formalise all gold mining activities through licensing, providing panners with permanent claims and operating permits in order to recoup some of the added costs in the form of taxes. Government, through the Ministry of Small Scale and Medium Enterprises, needs to improve assistance given to small-scale miners in the form of loans, safety clothing and machinery that can help improve their activities. Through the implementation of proper institutional frameworks artisanal small-scale miners can be held responsible and accountable for their activities.
